# Small bowel perforation due to ingested frog bone: a case report

**DOI:** 10.1093/jscr/rjae118

**Published:** 2024-03-07

**Authors:** Nguyen Dang Hung, Robert Michael O’Connell, Do Duc Minh, Michael Flanagan, Tran Bao Long

**Affiliations:** Department of General Surgery, Hanoi Medical University Hospital, Kim Lien, Hanoi, Vietnam; Institute of Global Surgery, Royal College of Surgeons in Ireland, Dublin D02 YN77, Ireland; Department of General Surgery, Hanoi Medical University Hospital, Kim Lien, Hanoi, Vietnam; Institute of Global Surgery, Royal College of Surgeons in Ireland, Dublin D02 YN77, Ireland; Department of General Surgery, Hanoi Medical University Hospital, Kim Lien, Hanoi, Vietnam

**Keywords:** small bowel perforation, bone ingestion

## Abstract

Perforation of the gastrointestinal tract by ingested foreign body is an uncommon surgical emergency, most typically associated with the consumption of fish and chicken bones. We present an unusual case of a gentleman presenting emergently with an acute abdomen following ingestion of a meal containing frog meat. Emergent computed tomography (CT) revealed findings suggestive of jejunal perforation due to a foreign body. At laparotomy, a mid-jejunal site of perforation was noted due to a protruding piece of fractured frog bone. Washout and primary repair of the small bowel enterotomy were performed, and the patient made an excellent post-operative recovery.

## Introduction

Perforation of the gastrointestinal tract by ingested foreign body is an uncommon occurrence, with only a small number of case series reported in the literature [1]. The majority of ingested foreign bodies will pass through the gastrointestinal tract without issue, however there is an associated risk of perforation, obstruction or bleeding in a minority of cases, which is increased in the presence of abnormal anatomy such as Meckel’s diverticulum or strictures [2]. The most commonly reported causative ingested foreign bodies are chicken and fish bones along with toothpicks [3]. There are no previously reported cases in the English language literature of an intestinal perforation related to ingestion of a frog bone.

## Case discussion

A 33-year-old gentleman was admitted to the Emergency Department of Hanoi Medical University Hospital with a 2-day history of severe central abdominal pain and new onset pyrexia. This had begun approximately 12 hours after consuming *lau ech*, a traditional Vietnamese shared dish containing frog meat.

His medical history was significant for peptic ulcer disease, managed with proton pump inhibitors. He denied any previous surgical history.

**Figure 1:**
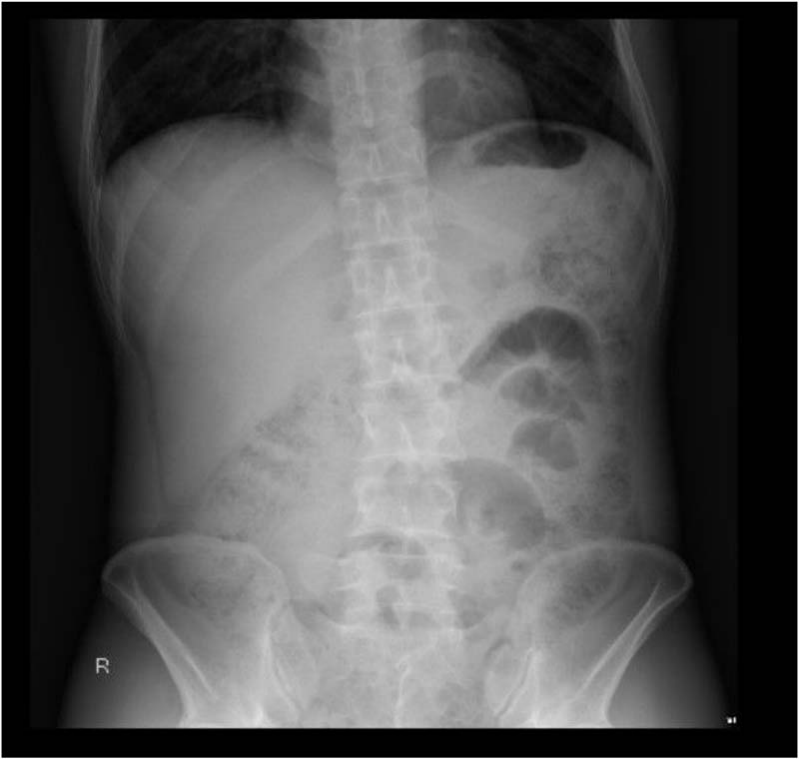
Abdominal X-ray with subtle evidence of pneumoperitoneum.

**Figure 2:**
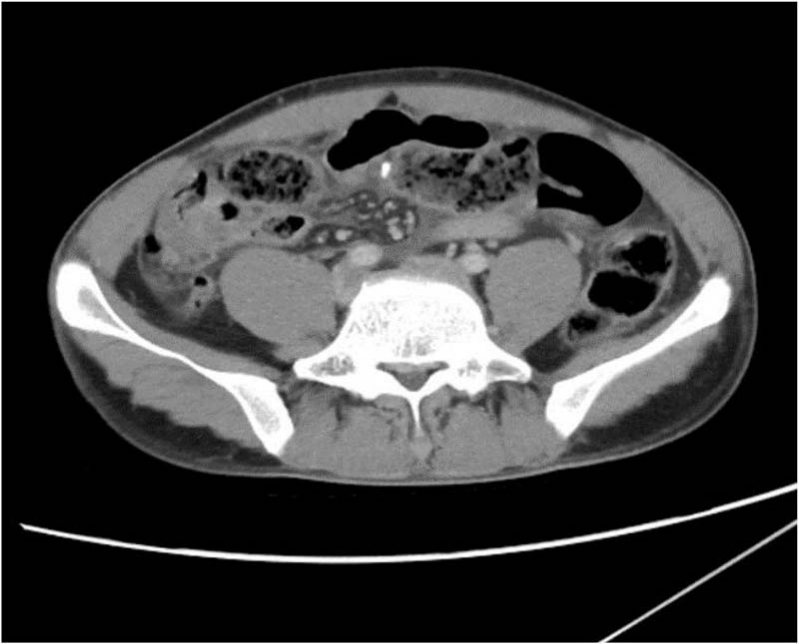
CT scan of abdomen and pelvis. A radiopaque object is seen extruding from a thickened small bowel loop with associated free peritoneal fluid, concerning for foreign body perforation of small bowel.

On initial presentation the patient was pyrexial, with a fever of 38.9 °C, but haemodynamically stable. Abdominal examination revealed diffuse peritonitis. Laboratory results showed a mild lecuocytosis (white cell count 10.5 g/L) but were otherwise within normal limits. Plain film X-ray of his abdomen was suggestive of pneumoperitoneum (see Fig. 1). A clinical diagnosis of suspected perforated duodenal ulcer was made and the patient was initially resuscitated with intravenous fluids and broad-spectrum antibiotics while a computed tomography (CT) scan of his abdomen and pelvis was arranged. The CT revealed showed a small rod-shaped radiopaque object in a thickened small bowel loop in the hypogastric region, with associated free abdominal fluid and air (see Fig. 2). This was concerning for small bowel perforation due to a foreign body.

**Figure 3:**
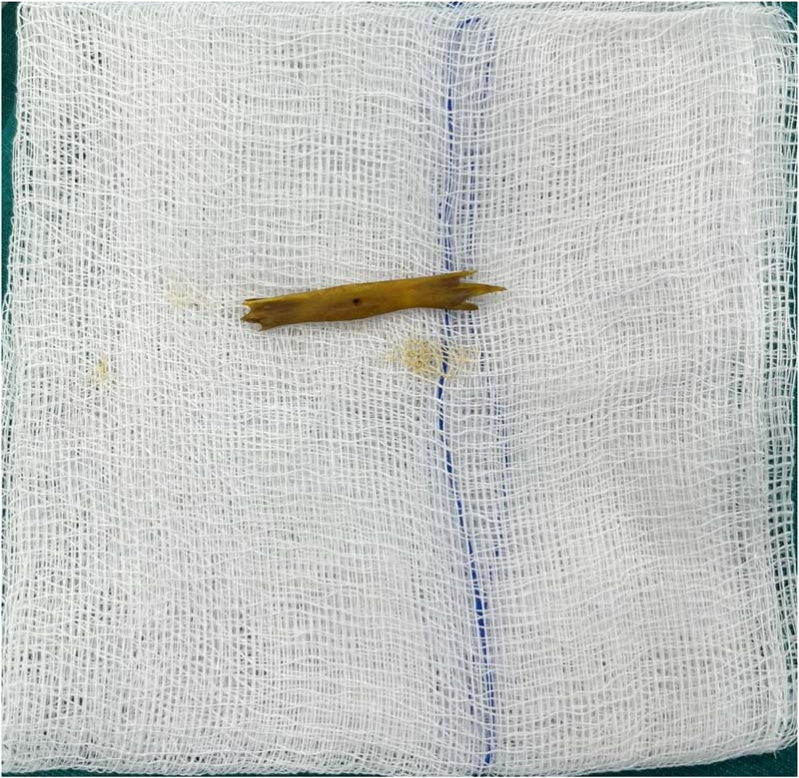
Fractured piece of bone following extraction.

**Figure 4:**
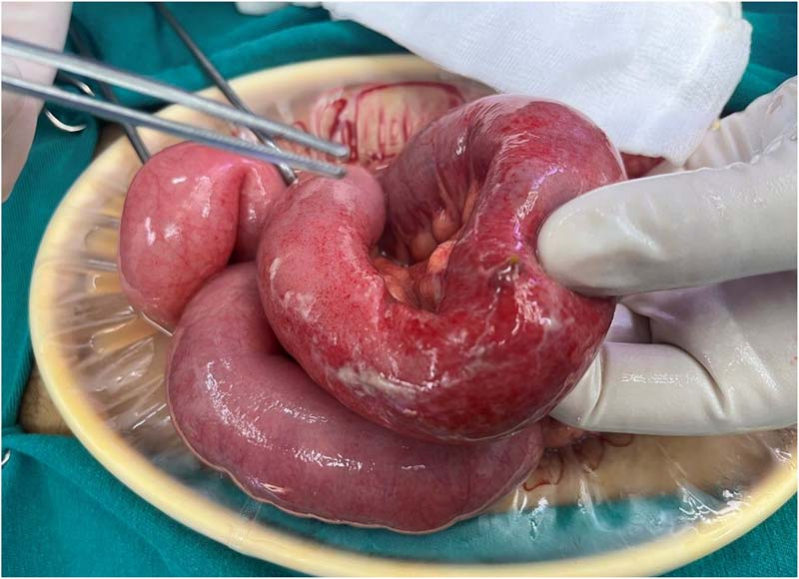
Site of perforation in mid jejunum.

Following imaging and discussion with the patient, we proceeded to emergency laparotomy. On evaluating the small bowel, an enterotomy was noted in the mid jejunum due to a protruding fractured piece of frog bone (see Figs 3 and 4). There was associated localised peritonitis, but no gross peritoneal contamination. A sample was taken for culture and sensitivity, but no growth was ultimately seen. A decision was made to proceed with a primary repair of the enterotomy as the surrounding small bowel tissue was healthy, and this was performed in two-layers with interrupted 4–0 PDS. An extensive abdominal washout was performed prior to closure. The patient made an uneventful post-operative recovery and was discharged home well on post-operative Day 7.

## Discussion

We present a case of an unusual cause of intestinal perforation. Indeed, few case reports exist in the English language literature of human pathology related to consumption of frog meat and we have not encountered any other reports of visceral perforation due to frog bone ingestion in the literature. Eating undercooked frog meat is reported to be a risk factor for developing sparganosis, a parasitic infection due to ingestion of larvae of the tapeworms belonging to the genus *Spirometra* in endemic regions such as Southern China [4].

Ingested foreign bodies will typically pass through the gastrointestinal tract without issue, but objects with sharp ends pose a risk for perforation, usually at angulated points within the gut, such as the ileocaecal valve, colonic flexures, or pathological or anatomical variants such as a stricture [5]. The bowel itself has protective adaptations to reduce the risk of perforation from sharp objects—a prick to the mucosal results in focal dilatation of the bowel facilitating passage of the object, which is generally conveyed through the gut with the broader, less sharp, end pointing forward due to peristalsis and the flow of enteric content [2].

The clinical presentation of patients with symptomatic ingested foreign bodies can vary greatly and can be hard to distinguish from more common acute abdominal presentations such as acute appendicitis or perforated duodenal ulcer [6]. As a result of this, radiological investigation forms the cornerstone of diagnosis. While many ingested foreign objects, including consumed animal bones, are radio-opaque and visible on plain X-ray, CT is generally considered to be the modality of choice to facilitate diagnosis, localisation and evaluation for complications such as perforation or fistulation [7].

Operative management is the treatment of choice for perforations due to foreign body perforation of the small bowel, while a careful multi-disciplinary approach may be required for those patients with localised complications such as fistulation into adjacent organs or vascular structures [8]. The decision to primarily repair the small bowel, as in this case, or to perform a segmental small bowel resection depends on surgeon preference, patient factors and the extent of small bowel injury, and both strategies are well described in the literature [9, 10].

## Conclusion

Ingested foreign bodies are an uncommon cause of small bowel perforation and the clinical presentation can mimic more common causes of acute abdominal pain. We present an unusual case of perforation due to ingestion of a frog bone, which was successfully managed with washout and primary small bowel repair at laparotomy.
